# Green mediated synthesis of cerium oxide nanoparticles by using *Oroxylum indicum* for evaluation of catalytic and biomedical activity

**DOI:** 10.1039/d4ra04132a

**Published:** 2024-08-13

**Authors:** Jannatul Mim, Mst. Sabiha Sultana, Palash Kumar Dhar, Md. Kamrul Hasan, Sagar Kumar Dutta

**Affiliations:** a Chemistry Discipline, Khulna University Khulna 9208 Bangladesh sagar@chem.ku.ac.bd; b Agrotechnology Discipline, Khulna University Khulna 9208 Bangladesh

## Abstract

The present perspective emphasizes the green synthesis of CeO_2_-NPs using *Oroxylum indicum* fruit extract. The synthesized NPs were characterized utilizing analytical techniques, including FT-IR, UV-vis, XRD, SEM-EDX, and VSM. Of them, XRD analysis ratifies the cubic fluorite crystal structure along with a particle size of 23.58 nm. EDX results support the presence of cerium and oxygen in a proper ratio. The surface morphology of NPs, however, was scrutinized using SEM. The lower IC_50_ value (20.8 μg mL^−1^) of NPs compared to the reference substance, ascorbic acid (33.2 μg mL^−1^), demonstrates CeO_2_-NPs to be a compatible antioxidant. Moreover, the drug-releasing capability of CeO_2_-NPs was a buffer pH-dependent parameter. The acidic pH solution was 20.5%, while the basic pH solution was 16.9%. The drug-releasing capability was analyzed using the Higuchi model and Korsmeyer–Peppas kinetics. The values of the determination coefficient (*R*^2^) were found to be 0.9944 and 0.9834, respectively. The photocatalytic activity of CeO_2_-NPs was evaluated, considering methylene blue as a model dye. The degradation percentage was attained up to 56.77% after it had been exposed for 150 min. Apart from this, the synthesized NPs were screened against two fungus species, *Bipolaris sorokiniana* and *Fusarium*. The percentage of growth was measured at 56% and 49%, respectively.

## Abstract 2

This perspective demonstrates the green synthesis of CeO_2_-NPs with *Oroxylum indicum* fruit extract.

## Graphical abstract

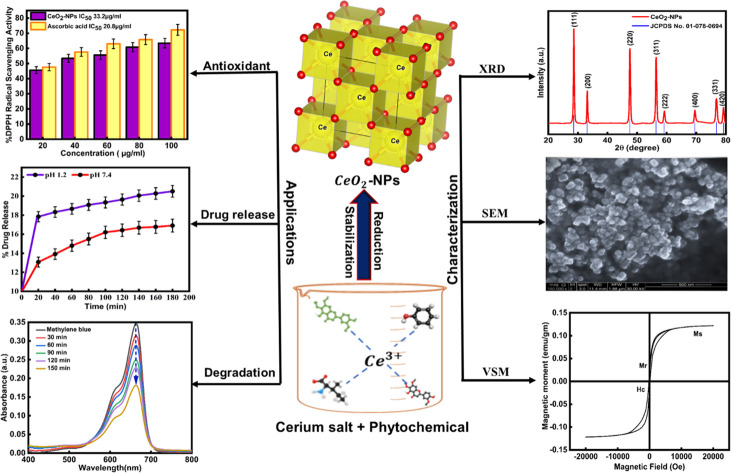

## 1. Introduction

Nanobiotechnology is a miracle of modern science that correlates material science with the scientific domain.^1,2^ However, nanobiotechnology employs nanosized particles with a diameter of 100 nm in biological processes, including antimicrobial agents, drug delivery, and nanomedicine.^3^ Nanoparticles are commonly used as nanocarriers in a variety of cost-effective fields, such as chemistry, physics, and mechanical engineering. In the medical sector, nanocarriers have attracted much attention because of their great degree of stability in the blood, non-immunogenicity, non-inflammatory, non-toxicity, and biodegradability. Nanocarriers also have the cost-effectiveness of synthesis methods, scalability, ease of use, and accessibility of a suitable alternative for chemical and physical preparation procedures.^4,5^ The use of nanocarriers has already arisen as a separate discipline that can facilitate biological sensing, cell labeling, targeting, imaging, and diagnostics.^6^ Metallic nanocarriers such as MgO, Fe_2_O_3_, TiO_2_, and ZnO comprise inorganic metal or metal oxide existing in the core position and have amassed significant attention owing to their tiny size, fingerprint structure, enlarged surface area, minimal toxicity, great capacity for cellular absorption, and lack of immune reaction.^7–9^ Cerium oxide (CeO_2_) NPs, a prominent nanocarrier, are extensively used in nanomedicine for drug dispensing, catalysis, biosensing, and medicinal applications.^8,9^

Metal oxide nanocarriers are usually synthesized using sol–gel, microwave, hydrothermal, biological parts, and co-precipitation techniques that are included as physical, chemical, and biological approaches.^10–12^ Recently, green synthesis, a type of biological method, has drawn the attention of scientists in nanotechnology due to the myriad sources of plants, microorganisms, and animals that are comparatively biologically active. Green synthesis requires less use of chemicals, is environmentally benign in nature, is easy to synthesize, and has low toxicity.^13–15^ Nature as a biological laboratory provides new species of fungi, bacterium cells, algae, and plants that possess various phytochemicals, including tannins, flavonoids, and terpenoids.^16^ Surprisingly, the phytochemicals function as reducing agents as well as stabilizing agents and capping agents of metal ions and nanoparticles, respectively.^16^ Moreover, the stabilizing agents impede nanocarriers from aggregation and provide various modes of bonding with nanoparticles.^17^ In this work, the green synthesis of cerium oxide nanoparticles from the aqueous extract of *Oroxylum indicum*, a plant species with medicinal properties, has been employed for the synthesis of bioactive CeO_2_-NPs.^18–20^*Oroxylum indicum* contains a wide range of bioactive substances that have been identified as potentially useful in the treatment of pneumonia, piles, cardiac conditions, diarrhea, and tannins, glycosides, saponins, phenols, and quines. In addition to these benefits, the compounds have made it easier to synthesize CeO_2_-NPs in an environmentally friendly manner. They also function as capping agents, stabilizing agents, and reduce toxicity while enhancing dispersion, stability, and prevention of agglomeration. Finally, the compounds maintain a higher surface area for catalytic activity and encourage a more controlled release of their antioxidant properties.^17,21^

There are two distinct oxidation states for cerium: tetravalent Ce^4+^ and trivalent Ce^3+^. Consequently, depending on the material's composition, cerium oxide can exist as two distinct oxides, such as Ce_2_O_3_ (Ce^3+^) and CeO_2_ (Ce^4+^). CeO_2_-NPs have a cubic fluorite crystal structure, and Ce^3+^ and Ce^4+^ coexist on their surface. CeO_2_-NPs have reducing reactive oxygen species (ROS) as an antioxidant activity. Those nanoparticles have a wide range of applications in different fields due to their two distinct oxidation states.^18,19,22^ Basically, the electropositive charge present in the surface cavities of CeO_2_-NPs enables them to interact with free charge-carrying species and lessen internal oxidative stress.^23^ These NPs have the potential to function as antimicrobial agents because they cause oxidative stress in fungi and bacteria. Another prominent application of CeO_2_-NPs was assessing the drug delivery approach along with Metronidazole (MTZ), an effective antimicrobial agent.^24–26^ It is surprising that in contact with aerobic bacteria, the antimicrobial agents exert negligible or no antibacterial activity. Furthermore, the drug has been classified as IV in the biopharmaceutical classification system (BCS) owing to its low permeability and incredibly low water solubility (1 mg mL^−1^), resulting in poor absorption and low bioavailability.^27,28^ This finding was certainly confirmed through the application of nanotechnology because of the alteration of physical and chemical properties.^29^ Beside theses, CeO_2_-NPs have significant antifungal activity, bolstered by various previous studies. Shahbaz *et al.* (2022) synthesized CeO_2_-NPs using *Acorus calamusas rhizomes* extract and screened against *the Puccinia striiformis* fungus, which revealed a positive response.^30^ In another study, Costa *et al.* (2023) conducted research against *Bipolaris sorokiniana*, a fungus that create root diseases in wheat.^31^

In the last few decades, environmental pollution has become a burning issue all over the world, and essential parts of the environment are frequently polluted through anthropogenic and natural process. The indiscriminate use of toxic materials, textile dyes, pesticides, and aromatic compounds is defiling the aqueous medium. The dumping of waste materials containing organic dyes can exert an adverse impact on biota and polluted water. To get rid of this impact, various analytical techniques have been initiated recently to remove or degrade the organic dye into less harmful chemical species. The available methods include chemical oxidation, photo-degradation, electrochemical oxidation, coagulation, flocculation, *etc.* The various limitations of the methods, such as lack of efficiency, complexity, and high energy consumption, make them comparatively less popular. On the contrary, NPs as photo-catalysts draw an appeal to eliminate the toxic dye from an aqueous medium owing to their reusability, efficiency, and visible light.

As a result, the co-precipitation technique was used in this study to create CeO_2_-NPs through green synthesis utilizing fruit extract from *Oroxylum indicum*. The elements found in the fruit extract of *Oroxylum indicum* were utilized as efficient reducing and capping agents. In an indirect mechanism, the plant extracts help to facilitate the oxidation of Ce^3+^ to Ce^4+^ by functioning as complexing agents instead of as traditional reducers. It also investigates these nano carriers' antioxidant activity. It was demonstrated that CeO_2_-NPs had photocatalytic characteristics as they may degrade methylene blue (MB) dye. Additionally, CeO_2_-NPs have antifungal efficacy against *B. sorokiniana* and *Fusarium* plant pathogens. Moreover, the aim of this work was to examine how metal oxide nanocarriers, especially CeO_2_-NPs, affected the loading and release of MTZ, a model drug.

## 2. Experimental

### 2.1 Chemicals and reagents

All chemicals and reagents used for the synthesis of CeO_2_-NPs, drug delivery, antioxidants, and dye degradation were purchased from Sigma-Aldrich and Merck. All chemicals and solvents were used directly without any further purification. Besides, double-deionized water (RCI Labscan Limited) was used throughout the whole study.

### 2.2 Preparation of plant extract

**Fig. 1:**
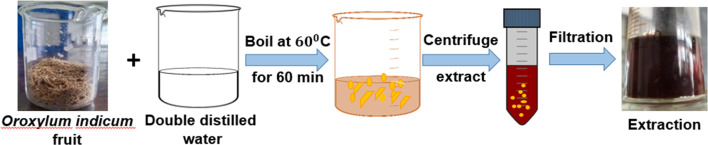
Diagram demonstrating the procedure for extracting phytochemicals.

*Oroxylum indicum* fruit was collected from the Khulna University campus, washed, and dried at ambient temperature. The fruit was ground, and an aqueous extract was made by mixing 50 mL of distilled water with a certain amount (5 g) of powdered plant fruit (10 : 1), and the mixture was boiled for 2 h at 60 °C until it turned into a bark-red extract. The extraction was allowed to cool to ambient temperature, and fruit extracts were obtained after centrifugation of the aqueous solution at 5000 rpm for 15 minutes (Fig. 1). The plant extract was kept at a low temperature (−18 °C) for further experimentation.^32,33^

### 2.3 Synthesis of CeO_2_ NPs

**Fig. 2:**
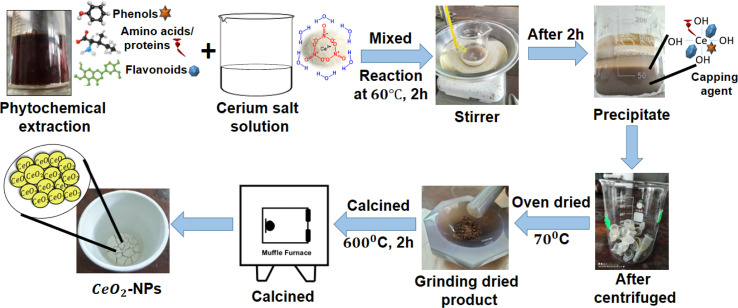
Diagrammatic representation of the environmentally friendly synthesis of CeO_2_-NPs.

The CeO_2_-NPs were synthesized using the following method, with slight modifications:. A certain amount of cerium(iii) nitrate hexahydrate salt (3.72 g) was dissolved in 10 mL of distilled water with constant stirring at room temperature for 30 min. The plant extract (10 mL) was added to the homogenous solution of cerium(iii) nitrate salt, and then the mixture was stirred for 2 h at 60 °C. The pH of the mixture was regulated using 1 M NaOH and adjusted to pH 11. The color of the solution changed to dark brown and formed a thick precipitation that was allowed to cool to ambient temperature. The mixture was centrifuged at 13 000 rpm for 10 minutes, washed with water three times, and dried in precipitate in an air oven at 70 °C overnight, resulting in a change of color to yellowish brown. The precipitate was calcinated at 600 °C for 2 h, and a yellowish-white precipitate was collected. As a result, powdered CeO_2_-NPs were created, and the chemical eqn (1) and (2) below reflect this. Additionally, a systematic diagram was given to explain the synthesis process shown in Fig. 2.^9,34^

1 Ce(NO_3_)_3_·6H_2_O_(S)_ + phytochemicles (green source)_(l)_ → Ce (OH)_3(S)_

2 4Ce(OH)_3(S)_ + O_2_ → 4CeO_2(S)_ + 6H_2_O_(l)_

### 2.4 Characterization of CeO_2_ NPs

The synthesized CeO_2_-NPs was characterized in several spectroscopic and analytical techniques, including UV-visible spectrophotometer (UV-1900i, SHIMADZU, Japan), Fourier Transform Infrared spectrophotometer (FT-IR) (IRSpirit, SHIMADZU, Japan) using a KBr pellet with a scan rate of approximately 4 cm s^−1^ at 25 °C, Scanning Electron Microscopy (SEM), Energy Dispersive X-ray spectroscopy (EDX) analysis (SU-8000, Hitachi, Japan) at accelerating voltages of 10 and 15 kV, and X-ray diffraction (XRD) (Rigaku Smart Lab spectrometer, Japan) with Cu-Kα radiation. The magnetic properties of NPs were analyzed using the Microsense VSM model EV7. A pH ION/EC/DO METER (MM-43X) was used to measure the pH of the solution.

### 2.5 Evaluation of *in vitro* antioxidant activity of green synthesized CeO_2_ NPs

The antioxidant capacity of CeO_2_-NPs at various concentrations, such as 20, 40, 60, 80, and 100 μg mL^−1^, was studied. An ethanoic solution of 2,2-diphenyl-1-picrylhydrazyl (DPPH) was prepared at a concentration of 25 μg mL^−1^. A fixed volume (2 mL) of different concentrations of CeO_2_-NPs was mixed with 2 mL of DPPH solution that was kept in the dark. After 30 min of incubation at room temperature, the absorbance of solution was measured at 517 nm. Ascorbic acid was used as a reference antioxidant compound. The percentage of inhibition was calculated using the following eqn (3).^9,35^3
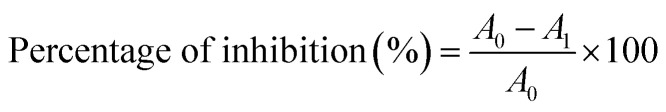
where *A*_0_ = absorbance of control and *A*_l_ = absorbance of treatments.

### 2.6 Preparation of MTZ-loaded CeO_2_-NPs

The CeO_2_-NPs suspension in the buffer solution (pH 1.2 and 7.4) was formulated at a concentration of 30 mg/40 mL *via* sonication. Two different pH solutions of 10 mg/10 mL MTZ followed the same process. The different pHs of the suspension were slowly added to the solutions of MTZ. The mixtures were sonicated for 3 hours. At regular intervals, the remaining concentration of MTZ in solution was measured using the UV-visible spectroscopic technique (SHIMADZU UV-1900i) at a wavelength of 320 nm. This allowed us to quantify the drug loading on the surface of CeO_2_-NPs. The nanoparticles were separated from the mixture before the measurement. Subsequently, it underwent centrifugation for ten minutes at a speed of 10 000 rpm. The white-colored slurry was earned. Following that, the gathered slurry was dried for 4 h at 50 °C in an oven and kept in a desiccator until testing.^36–38^

### 2.7 Estimating the drug loading capacity and the entrapment efficiency

CeO_2_-NPs were centrifuged at 12 000 rpm for 5 minutes to extract the drug loading capacity (DLC) and the drug entrapment efficiency (DEE). The absorbance of all samples was measured at 320 nm using UV-visible spectrophotometer.^36^ The loading capacity and entrapment efficiency percentages of the drug were measured using eqn (4) and (5), respectively.4
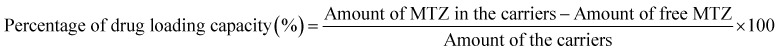
5
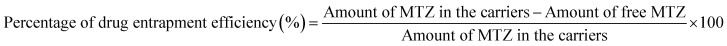


### 2.8 *In vitro* MTZ drug release study of CeO_2_-NPs

**Fig. 3:**
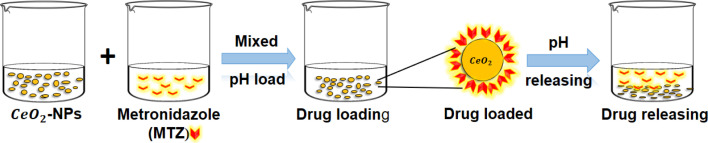
A schematic representation of the loading and release of CeO_2_-NPs using metronidazole (MTZ).

A disparity method was involved to evaluate MTZ release from CeO_2_-NPs (Fig. 3). A certain amount (20 mg) of MTZ-loaded CeO_2_-NPs was dissolved in 50 mL of different buffer medium at pH 1.2 and pH 7.4 at 37 °C. The CeO_2_-NPs were separated after 4 mL of the drug-released medium was removed and centrifuged at predetermined intervals. The amount of the released drug in the solution was measured using a UV-vis spectrophotometer at *λ*_max_ = 320 nm from 0 to 3 h. The percentage release of MTZ was calculated by the following eqn (6).^39–41^6
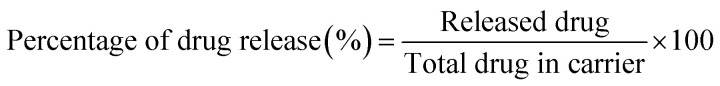


### 2.9 Photocatalytic activity green synthesized CeO_2_-NPs

The photocatalytic activity of CeO_2_-NPs was evaluated, considering methylene blue (MB) as a model dye. In a standard experiment, a fixed amount (6 mg) of photo-catalyst was added to 100 mL (10^−5^ M) of MB dye. The dye solution was magnetically stirred for 45 minutes in the dark to ascertain adsorption or desorption equilibrium. The photocatalytic process was initiated at intervals of 30 min by turning on the UV radiation. The absorbance of the solutions was measured using a UV-vis spectrophotometer at 664 nm. The percentage of photocatalytic degradation was evaluated using the following eqn (7).^42^7
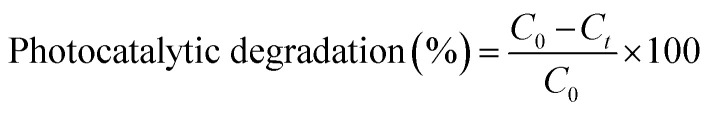
where, *C*_0_ is the initial concentration and *C*_*t*_ is the concentration after various time intervals (min).

### 2.10 Antifungal activity of CeO_2_-NPs

Antifungal activity of green synthesized CeO_2_-NPs was evaluated at 1.35 mg mL^−1^, and the solution was added to Petri dishes with PDA culture medium. The evaporation of ethanol and the diffusion of nanoparticles took 30 minutes, and after that, a disc of 5 mm in diameter of the corresponding strain mycelium (collected) was placed in the center of the PDA dish, which was then incubated at 28 °C. In addition, an absolute control solution (PDA + CeO_2_-NPs). The effect of CeO_2_-NPs was determined by measuring the diameter of the colonies after 5 days and calculating the percentage inhibition of mycelial growth by following eqn (8).^43^8
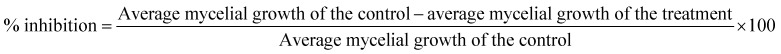


## 3. Results and discussion

In this report, an aqueous extract of *Oroxylum indicum* fruits to synthesise CeO_2_-NPs is presented. The multi functionality of *Oroxylum indicum* aqueous extract (*i.e.*, which acts as both reducing and capping agents). Green reducing agents are crucial during the process because they act as capping and stabilizing agents, which ensures that the generated NPs have the appropriate sizes, shapes, and fewer agglomeration behaviors.

### 3.1 Characterization of NPs

#### 3.1.1 Electronic absorption spectroscopy

**Fig. 4:**
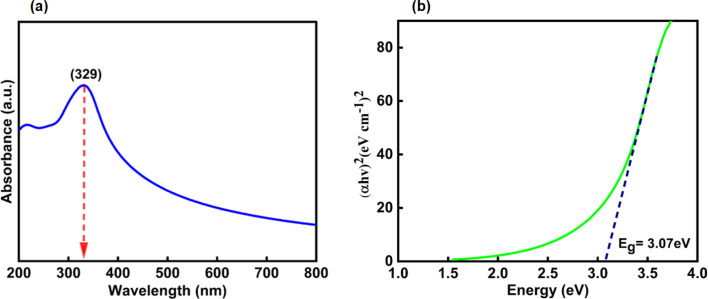
Shows (a) UV-visible spectrum and (b) band gap of CeO_2_-NPs.

The UV-visible absorption spectrum of CeO_2_-NPs and *Oroxylum indicum* fruitshows extract has been illustrated in Fig. 4(a), and the absorbance was measured ranging from 200 to 800 nm. The strong absorbance was observed at 329 nm, ascertaining characteristic peak of CeO_2_-NPsthat was quietly absent in plant extract. The UV absorption edge was used to precisely forecast the band gap of any system, as shown in Fig. 4(b). The optical energy band gap for CeO_2_-NPs was calculated using Tauc's eqn (9).^44^9
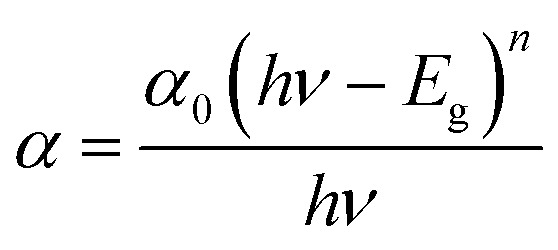
where *hν* is the photon energy, *α* is the absorbance coefficient, *α*_0_ is the characteristic parameter, h is the Planck's constant, and *n* is the power factor. Depending on the nature of the transition, the value of *n* may vary, and it is to be 2. The band gap, *E*_g_, for CeO_2_-NPs was calculated at 3.07 eV, indicating that it is comparatively higher than bulk cerium. The presence of organic compounds on the nanoparticle surface elevated the band gap as well as good absorption at 329 nm, suggesting applications in various fields.^45^

#### 3.1.2 FTIR analysis

**Fig. 5:**
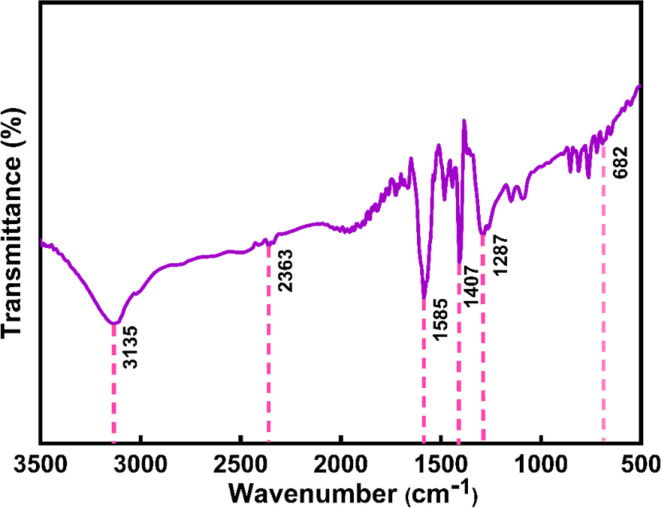
FTIR spectrum of (a) *Oroxylum indicum* fruit extract, and (b) CeO_2_-NPs.

The FTIR spectrum for the extract of *Oroxylum indicum* would display distinctive peaks that would correspond to the different functional groups that are present in the extract. The primary bands were seen in Fig. 5(a) at 3324, 1647, 1516, and 1092 cm^−1^. The O–H is indicated by the peak at 3200–3600 cm^−1^, which may be caused by the presence of alcohol groups and phenols. The bands at 1700–1600 cm^−1^ confirm the presence of an aromatic ic carbonyl group; 1516 cm^−1^ indicates C=C; and 1300–1000 cm^−1^ corresponds to C–O. Some weak absorption bands were also detected in the spectra of added ion.^46^ The presence of phytochemicals in *Oroxylum indicum* plant extract contributed to the great extent of the stability of nanoparticles, and the prominent functional groups associated with phytochemicals were identified in the FT-IR spectrum illustrated in Fig. 5(b). The broad peak in the spectrum at 3135 cm^−1^ is attributed to the stretching vibration of the hydroxyl group (–OH), and additionally, the characteristic peak at 1585 cm^−1^ is an indication of the C=C bond in organic compounds.^9^ The signature peak of this spectrum, at 692 cm^−1^, reveals the formation of a Ce–O bond.^34,36^

#### 3.1.3 SEM and EDX analysis

**Fig. 6:**
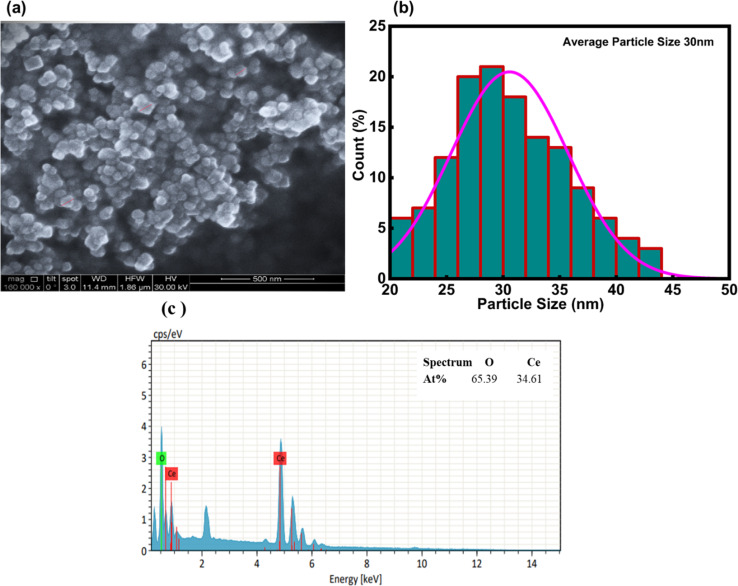
(a) Shows the SEM of CeO_2_-NPs, (b) the average particle size distribution graph in nm, and (c) EDX analysis.

Field emission scanning electron microscopy was employed to examine the surface morphology, arrangement, and purity of the nanoparticles. Fig. 6(a) presents the formation of individual nanoparticles without any aggregation. Fig. 6(b) depicts the particle size distribution, eliciting fairly consistent particle morphology. According to the particle size analysis curve, the average particle size of CeO_2_-NPs was found to be 30 nm. The elemental composition of NPs was evaluated using energy dispersive X-ray (EDX) spectroscopy. The percentage of elements found in the EDX spectrum bolsters the confirmed nanoparticles' purity. Analysis of CeO_2_-NPs, as seen in Fig. 6(c), confirmed the presence of oxygen and cerium in a mixed condition.

#### 3.1.4 XRD analysis

**Fig. 7:**
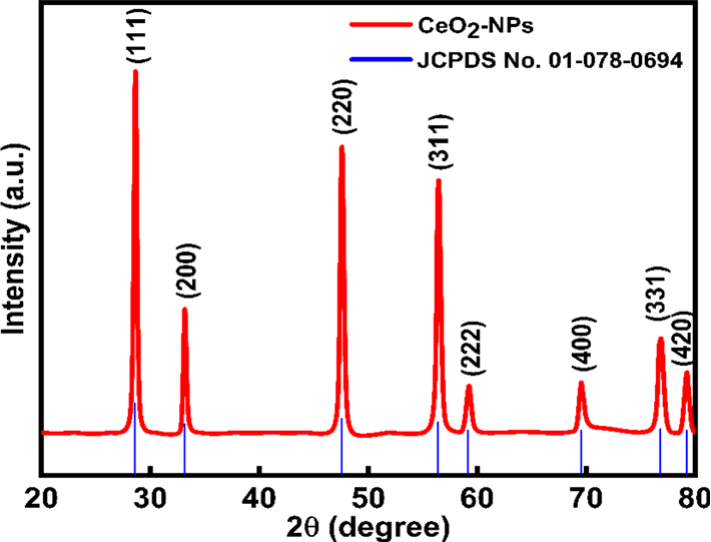
XRD results of the CeO_2_-NPs.

This analytical approach was utilized to ascertain the distinct crystalline phases of binary metal oxide nanoparticles. Fig. 7 displays the XRD pattern of green synthesized CeO_2_-NPs. The NPs possessed the cubic fluorite crystal structure, and the XRD pattern revealed the presence of cerium oxide phases. The diffraction angle (2*θ*) at 28.58°, 33.12°, 47.54°, 56.41°, 59.16°, 69.50°, 76.80°, and 79.18° corresponded to (111), (200), (220), (311), (222), (400), (331) and (420) planes, respectively, supported by JCPDS no. 01-078-0694.^44^ The lattice parameters, including unit volume, crystallite size, micro-strain, and dislocation density of CeO_2_-NPs, have been represented in Table 1.^47^

**Table 1:** Different parameters of CeO_2_-NPs from XRD

Sample name	Parameters	Equation	Equation number	Ref.
CeO_2_-NPs	Lattice parameter, *a* (nm)	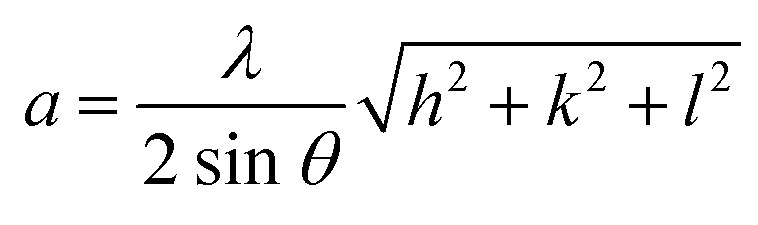	(10)	47
	Unit volume (*a*^3^) nm^3^	*V* = *a*^3^	(11)	48
	Average crystallite size (*D*) nm	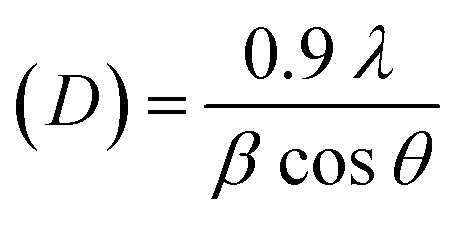	(12)	9
	Micro strain (*ε*) × 10^−3^	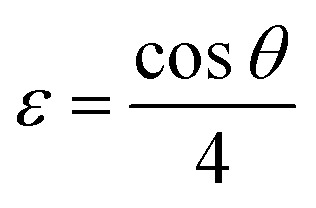	(13)	47
	Dislocation density (*δ*) × 10^−3^	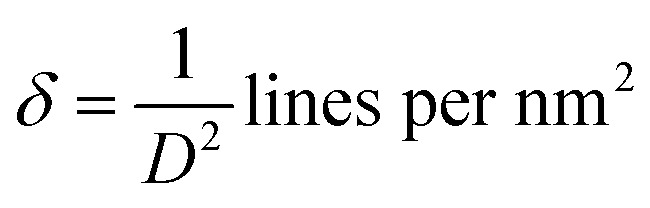	(14)	47

The lattice parameter “*a*” was estimated as 5.405 Å (0.5405 nm) for synthesized NPs. It possessed an exceptional degree of crystallinity and a single-phase cubic fluorite crystal structure inside the (*Fm*3̄*m*) space group. Besides, the unit volume of produced green NPs was found to be 157.901 Å^3^ (0.157901 nm^3^), calculated by using the following eqn (11).^48^ The average crystallite size of CeO_2_-NPs was calculated using the Debye–Scherrer formula shown in eqn (12).^9^ Where *D* is the crystallite size, *λ*-is the X-ray wavelength of CuKα radiation, *β*-is the full-width half maximum of the peak, and *θ* represents Bragg's diffraction angle. The average crystallite size of green synthesized CeO_2_-NPs was calculated at 23.58 nm. Another important parameter, such as the micro-strain of CeO_2_-NPs, was calculated using eqn (13).^47^ It is noted that the calculated micro-strain of CeO_2_-NPs was 1.48 × 10^−3^, and a crystallographic defect infers to an imperfection in the crystal structure. The dislocation density of CeO_2_-NPs was mathematically calculated using the above-listed model eqn (14).^48^ Where 1.79 × 10^−3^ is the dislocation density of the green synthesized CeO_2_-NPs, which shows the electrochemical and catalytic characteristics. In addition, different papers reported that different sources were used for the synthesis of CeO_2_-NPs. Those NPs have a comparative value of XRD parameters with this work, and Table 2 shows this comparison.

**Table 2:** Presents a comparison of the XRD parameters of CeO_2_-NPs synthesized from *Oroxylum indicum* with those from different sources

Different sources of synthesis CeO_2_-NPs	Lattice parameter, *a* (nm)	Unit volume (*a*^3^) nm^3^	Average crystallite size (*D*)nm	Micro strain (*ε*) × 10^−3^	Dislocation density (*δ*) × 10^−3^	Ref.
*Gloriosa superba* L. leaf extract	0.5416	0.158867	24	—	1.73	48
*Salvadora persica* bark extract	0.5431	0.16019	5.66	0.86	0.312	49
Chemically	0.5404	0.15808	18.66	4.1	6.74	44
Chemically	0.5450	0.161878	10.98	2.9105	8.2945	47
*Oroxylum indicum* fruit extract	0.5405	0.157901	23.58	1.48	1.79	This work

#### 3.1.5 Rietveld refinement

**Fig. 8:**
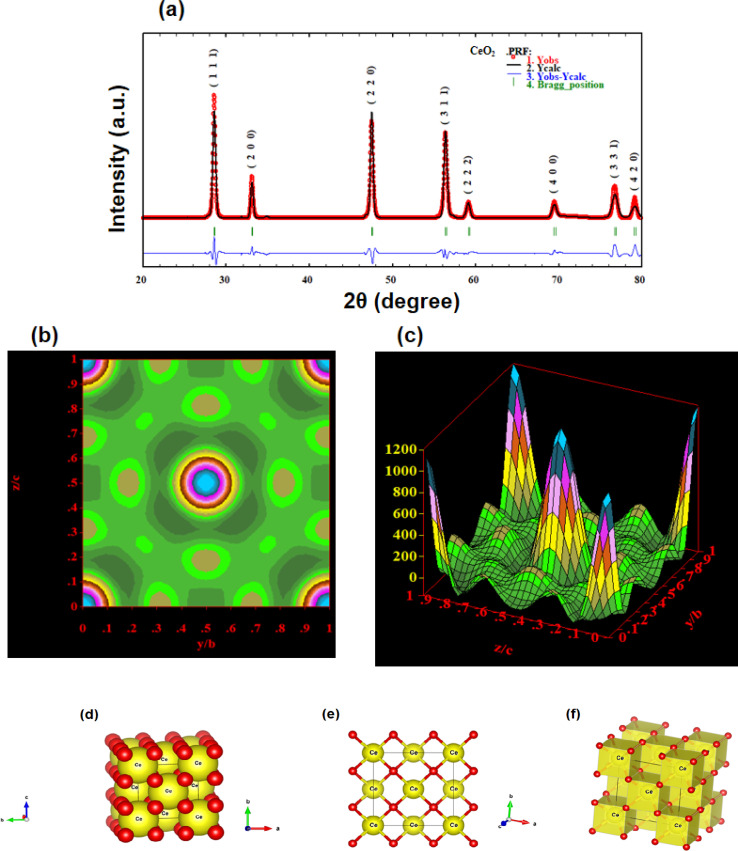
Shows the (a) Rietveld refinement graph; (b) electron density mapping in 2D counter form; (c) electron density mapping in 3D form; (d) crystal structure of CeO_2_-NPs in the single unit cell; (e) crystal structure of CeO_2_-NPs in a single layer; and (f) crystal structure in a polyhedral structure.

CeO_2_-NPs were refined using the Rietveld method with the aid of the Full-Prof Suite application and Vista software. Fig. 8(a) shows the refinement plot of CeO_2_-NPs. Straightforwardly, the findings of an X-ray structural determination are electron density maps. Essentially, electron density maps are the results of an X-ray structural determination. The electron density mapping, which shows the locations of electrons, oxygen, and cerium, is explained in Fig. 8(b) and (c). The maps show how well the structural model fits the data that was collected through empirical means. By using Vista software and XRD data, the crystal structure was created, as seen in Fig. 8(d)–(f). The crystal structure of cerium oxide is now visible in Fig. 8(d). To illustrate the locations of the atomic layers, we will show a single layer of cerium oxide in Fig. 8(e).^44^Fig. 8(f) is the final representation of the polyhedral structure of cerium oxide. In addition to having 78 atoms, 112 bonds, and 14 polyhedral units, figures show the unit cell structures of CeO_2_.

#### 3.1.6 VSM analysis

**Fig. 9:**
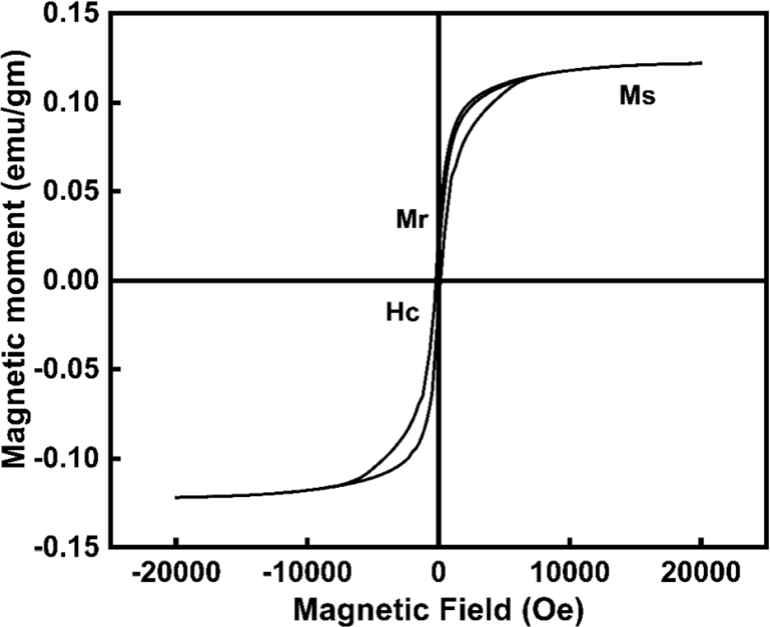
Emphasize the VSM of CeO_2_-NPs.

The magnetic properties of CeO_2_-NPs were assessed using VSM analysis, and Fig. 9 illustrates the M–H curve of CeO_2_-NPs recorded at room temperature. At normal temperatures, CeO_2_-NPs showed ferromagnetic properties owing to presence of oxygen vacancies.^50^ The hysteresis loop (*H*_c_) was observed, with a coercive of 200 Oe at 600 °C (23 nm size). 0.12 emu gm^−1^ of saturation magnetization (*M*_S_), and 0.023 emu gm^−1^ of retentivity (*M*_r_). In this study, CeO_2_-NPs exhibit ferromagnetic properties. Due to a corresponding number of Vo compensating for the reduction in positive charge caused by Ce^3+^. Changes in particle size have an impact on the development of surface defects such as Vo, which can exist on the particle surface in the Ce^3+^ or Ce^4+^ state. According to some theories, ferromagnetism in nanostructures might be enhanced by oxygen vacancies (Vo).^51,52^ On nearby Ce^3+^ ions (Ce^3+^–Vo–Ce^3+^), the oxygen vacancies may directly promote ferromagnetic coupling.^50^

#### 3.1.7 Estimation of the surface charge of CeO_2_-NPs

Here, there are electrostatic forces between the ionic species of the pollutant molecules and charges on the surface of any catalyst. Thus, depending on the pH of the aqueous solution, hydroxyl anions or proton cations can be adsorbed on the solid surface and used as a catalyst or an adsorbent. As a result of the solid particles' basic future, or native negative, and the solution's tendency to become more alkaline, there were fewer protons. Conversely, the solid particles in acidic solutions cause them to attract hydroxyl anions from the contracted solution at a pH higher than pHpzc. When the transition from the native basic future to the acidic future starts, the solid catalysts are neutral at pHpzc.^53,54^

**Fig. 10:**
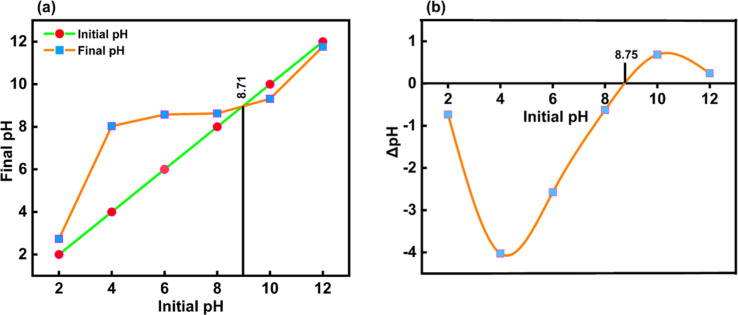
(a and b) Shows typical plots used to determine the pHpzc of CeO_2_-NPs.

The pHpzc was determined by graphing the starting pH (pH_I_) of the suspensions against their final pH (pH_F_) in Fig. 10(a), resulting in the bisector displayed. Before pHpzc, the suspension's final pH was higher than pHpzc. For CeO_2_-NPs, the pHpzc value was 8.71 at the point where this curve crossed the bisector. Plotting this curve, ΔpH (the difference between pH_I_ and pH_F_), against pH_I_ is shown in Fig. 10(b). The pHpzc value is the point on the curve where ΔpH is zero, or the point where the curve crosses the *x*-axis. According to both plots, the pHpzc of synthesized CeO_2_-NPs is roughly 8.7.^55^

### 3.2 Antioxidant activity green synthesized CeO_2_-NPs

**Fig. 11:**
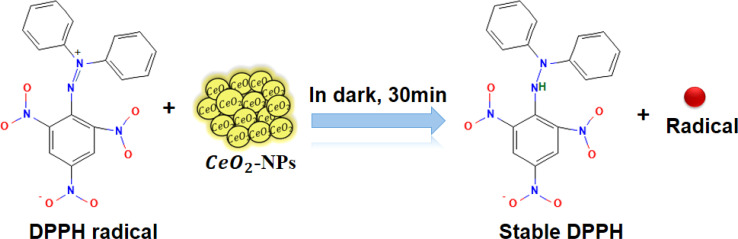
Schematic mechanism of DPPH scavenging by CeO_2_-NPs.

**Fig. 12:**
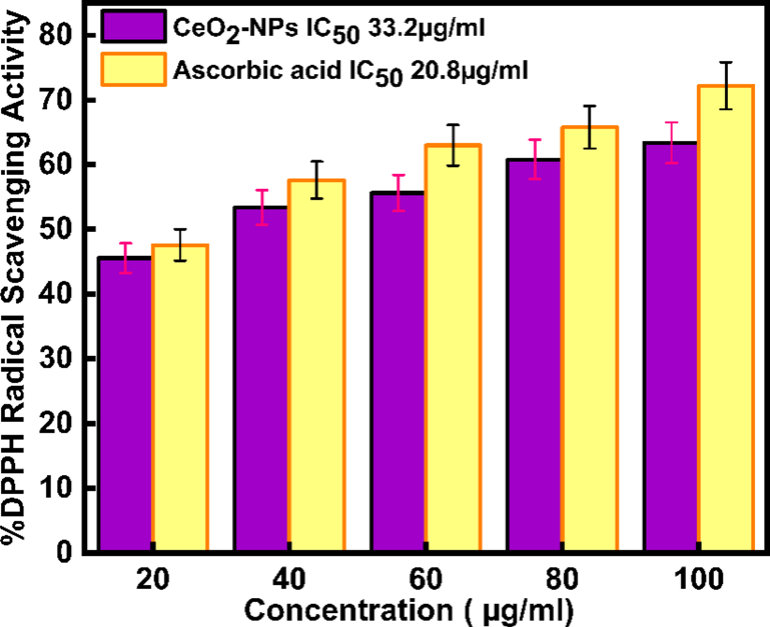
shows DPPH radical scavenging action of CeO_2_-NPs and ascorbic acid.

Today, in biological systems, the antioxidant activity of nanoparticles is a vital issue. Free radicals contain one or more unpaired electrons, produced due to the interaction between molecular oxygen and biomolecules in biological systems. These radicals can stabilize the unstable molecules in biology that are causing damage.^56^ To evaluate the antioxidant potential of green-synthesized CeO_2_-NPs using the DPPH antioxidant assay technique. The antioxidant activity of CeO_2_-NPs, redox recycling between surface Ce^3+^ and Ce^4+^, scavenging of free radicals, and Ce^3+^ ions are mainly involved.^57^Fig. 11 schematic mechanism of DPPH scavenging by CeO_2_-NPs. As Fig. 12 illustrates, the antioxidant activity of CeO_2_-NPs in different concentrations depends on the slow increase in nanoparticle concentration from 20 to 100 μg mL^−1^. For CeO_2_-NPs, at a high concentration of 100 μg mL^−1^, scavenging activity was recorded with percentages of 63.4 ± 3.17%. Compared to ascorbic acid (control), which recorded a scavenging activity of 72.2 ± 3.61%. The scavenging percentages decreased at low concentrations (20 μg mL^−1^), 45.6 ± 2.28%, and 47.6 ± 2.38% for CeO_2_-NPs and ascorbic acid, respectively. Similarly, CeO_2_-NPs-fabricated *Hyphaene thebaica* fruit extract showed antioxidant activity detected by DPPH scavenging methods with a value of 36.07% compared to ascorbic acid 87.89% at the same concentration of 400 μg mL^−1^.^58^ Also, CeO_2_-NPs fabricated from *Mentha royleana* plant extract detected antioxidant activity by DPPH scavenging methods with a value of 31% compared to ascorbic acid at 46%.^23^ Further, *Sargassum wightii* Greville-mediated CeO_2_-NPs showed maximum DPPH scavenging of 86.4% compared to ascorbic acid at 92.6% at the same concentration of 100 μg mL^−1^.^59^

The IC_50_ values in Table 3 for ascorbic acid and CeO_2_-NPs were measured at 20.8 μg mL^−1^ and 33.2 μg mL^−1^, respectively. As per the report by,^9^ the ascorbic acid and CeO_2_-NPs had IC_50_ values of 9.36 μg mL^−1^ and 15.47 μg mL^−1^, respectively.

**Table 3:** Antioxidant activity of *Oroxylum indicum*-mediated CeO_2_-NPs

Concentration μg mL^−1^	% DPPH radical scavenging activity	
	CeO_2_-NPs	Control
20	45.6 ± 2.28	47.6 ± 2.38
40	53.4 ± 2.67	57.6 ± 2.88
60	55.6 ± 2.78	63 ± 3.15
80	60.8 ± 3.04	65.8 ± 3.29
100	63.4 ± 3.17	72.2 ± 3.61

### 3.3 Drug loading and pH-responsive drug release of MTZ from CeO_2_-NPs

**Fig. 13:**
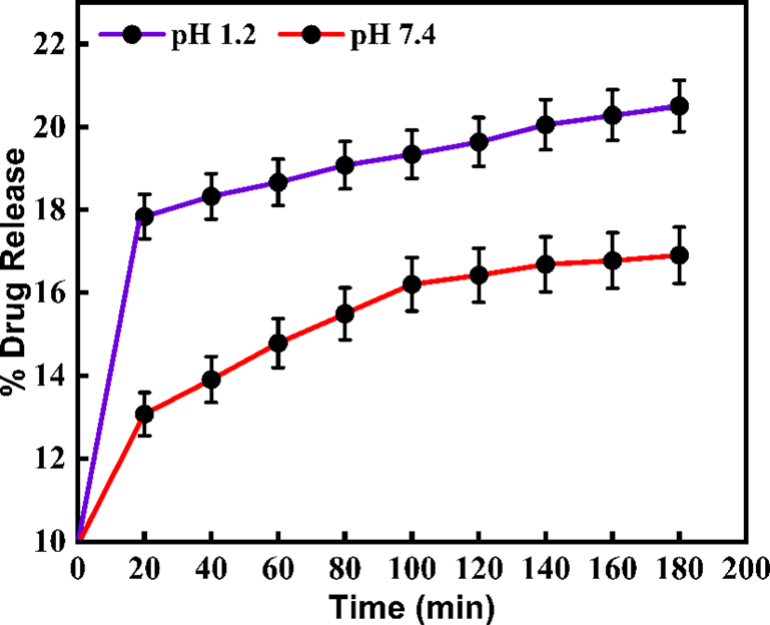
Shows the release profile at different pH solutions of MTZ-loaded CeO_2_-NPs.

The synthesized CeO_2_-NPs have drug-loading capability at different pHs. Metronidazole was a targeted drug. This drug was used for the pH-responsive release of the MTZ from the CeO_2_-NPs nano-drug delivery system. To determine the drug release profiles from the MTZ-loaded CeO_2_-NPs at different pH buffer solutions that were continuously recorded by UV-vis spectroscopy for the evaluation of the drug concentration. Further, using a mathematical method to calculate the drug loading capacity (DLC) and the drug entrapment efficiency (DEE) at different pH buffer solutions of CeO_2_-NPs. Based on outcomes, the DLC% of MTZ for CeO_2_-NPs at acidic buffer pH 1.2 was 1.13% and at basic buffer, pH 7.4 was 0.96%. Also, the DEE% for MTZ-loaded CeO_2_-NPs was determined to be the same buffer solution; pH 1.2 and pH 7.4 were found to be 72% and 58%, respectively. Based on release results, it is stated that, at acidic buffer pH 1.2, MTZ-loaded CeO_2_-NPs have a higher release (%) value than basic buffer at pH 7.4. These values were 20.5% and 16.9% at pH 1.2 and pH 7.4, respectively.^36^Fig. 13 reveals the release profile at different pH solutions of green synthesized CeO_2_-NPs.

### 3.4 Application of kinetic models

Table 4 shows how the different kinetic models were calculated using the coefficient of determination (*R*^2^) value from linear regression through eqn (15)–(18). These models included zero-order, first-order, Higuchi, and Korsemeyer–Peppas models.^60^

**Table 4:** Different kinetic models

Equation no.	Model	Equation	Ref.
(15)	Zero-order model	*Q* = *Q*_0_ + *kt*	60
(16)	First-order model	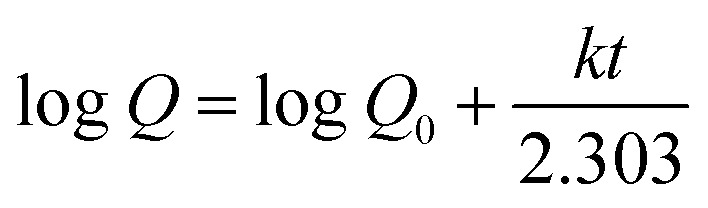	60
(17)	Higuchi model	*Q* = *kt*^0.5^	60
(18)	Korsmeyer–Peppas model	log *Q* = log *k* + *n* log *t*	60

Where, *Q* is the amount (mg mL^−1^) of the drug released at time *t*, *Q*_0_ is the initial amount of the drug in solution (it is usually zero), *t* is the release time (min), and *k* is a constant (mg mL^−1^ min^−1^) reflecting the geometric and structural characteristics of the carrier. According to the first-order release model, the plot of log *Q versus* time would give a straight line with a slope of *k*/2.303 (min^−1^). Moreover, Higuchi model represents the drug release kinetics model loaded into semisolid and solid matrices. Here, the concentration of the drug released is correlated to the square root of time with the slope of *k* (mg mL^−1^ min^−1/2^) The Korsmeyer–Pappas model describes the release of a drug from carriers of Fickian diffusion (Case I) (*n* ≤ 0.43), (non-Fickian) (0.43 < *n* < 0.85), and (Case II) transport (*n* ≥ 0.85), which describes the drug release mechanism. The value of *k* (mg mL^−1^ min^−1/*n*^) and *n* are obtained from the intercept and slope of the plot of log *Q versus* log *t*, respectively.

**Fig. 14:**
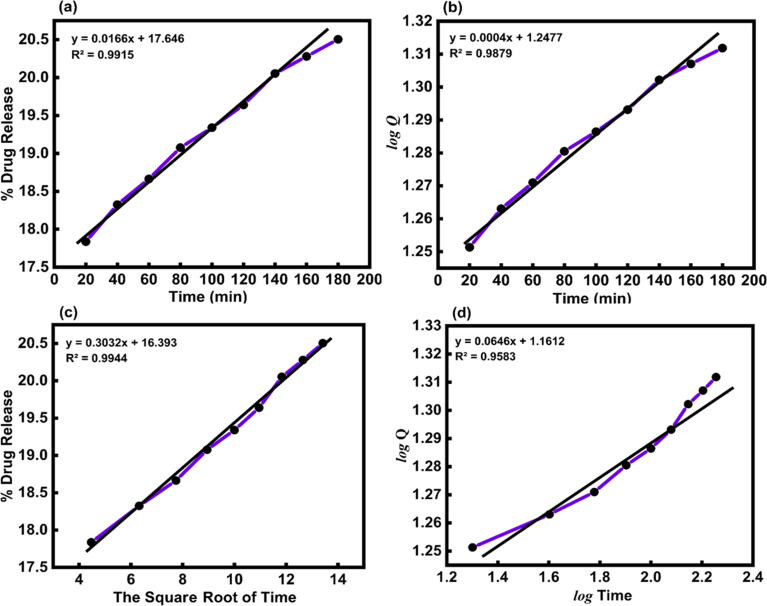
(a) Zero-order, (b) first-order, (c) Higuchi, and (d) Korsmeyer–Peppas kinetic models for the release of MTZ from CeO_2_-NPs at pH 1.2.

**Fig. 15:**
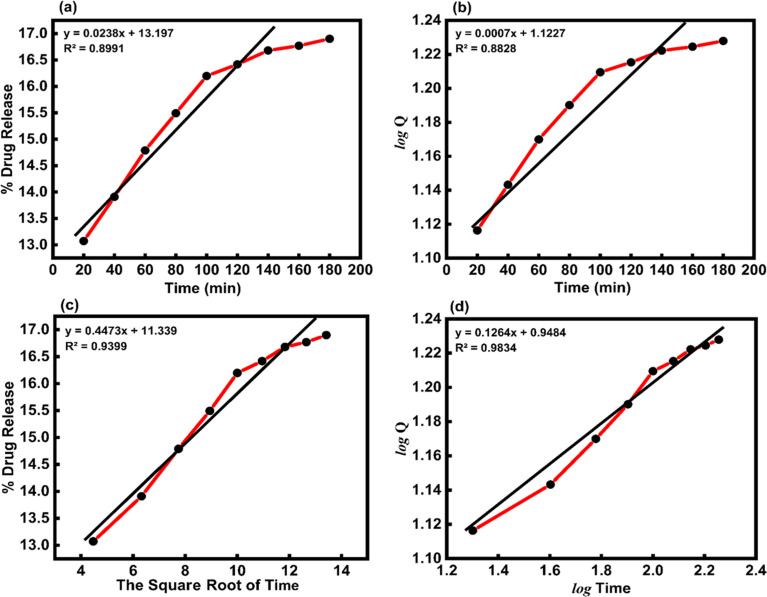
(a) Zero-order, (b) first-order, (c) Higuchi, and (d) Korsmeyer–Peppas kinetic models for the release of MTZ from CeO_2_-NPs at pH 7.4.

*In vitro*, the drug release kinetic were analyzed using various models and equations, including zero-order, first-order, Higuchi, and Korsemeyer–Peppas models (Table 5). To determine the kinetic model of the MTZ release from CeO_2_-NPs release, the obtained release data (until 180 min) were fitted to the above-mentioned equations. The obtained plots at pH 1.2 and pH 7.4 are depicted in Fig. 14 and 15.

**Table 5:** The kinetic parameters for the release of MTZ-loaded CeO_2_-NPs at different pH solutions

Kinetics model	Constants		Correlation coefficients (*R*^2^)	
	pH 1.2	pH 7.4	pH 1.2	pH 7.4
Zero-order model	*k* = 0.0166 (mg mL^−1^ min^−1^)	*k* = 0.0238 (mg mL^−1^ min^−1^)	0.9915	0.8991
First-order mode	*k* = 0.0004 (min^−1^)	*k* = 0.0007 (min^−1^)	0.9879	0.8828
Higuchi model	*k* = 0.3032 (mg mL^−1^ min^−1/2^)	*k* = 0.4473 (mg mL^−1^ min^−1/2^)	0.9944	0.9399
Korsmeyer–Peppas model	*k* = 1.1612 (mg mL^−1^ min^−1/*n*^), *n* = 0.0646	*k* = 0.9484 (mg mL^−1^ min^−1/*n*^), *n* = 0.1264	0.9583	0.9834

The Higuchi model kinetics equation for acidic buffer pH 1.2 yielded the highest value of the determination coefficient (*R*^2^), which was 0.9944. The Korsmeyer–Peppas kinetic model was the most appropriate for basic buffer pH 7.4, and its coefficient (*R*^2^) was 0.9834. When it comes to drug release systems with one-dimensional diffusion, the Higuchi model offers a foundation for comprehending the kinetic mechanism. As we looked at the *in vitro* MTZ release CeO_2_-NPs results, we saw that the correlation coefficients (*R*^2^) of the Higuchi model of MTZ release CeO_2_-NPs at pH 1.2 were close to 1. This means that the model fits the data the best. Furthermore, the value of the correlation coefficients (*R*^2^) of the Korsmeyer–Peppas kinetic models of MTZ release CeO_2_-NPs at pH 7.4 was found to be close to 1, and the calculated diffusional exponent “*n*” for the release of MTZ in CeO_2_-NPs by the Korsmeyer–Peppas model is 0.1264. According to Korsmeyer–Peppas results *n* < 0.43, it was proved that the release mechanism is controlled by Fickian diffusion for CeO_2_-NPs. The drug molecules in this investigation are bonded to and adsorbed onto the active regions of the nanoparticles.^61,62^ In addition, different papers reported that MTZ drug release kinetics by nano carriers followed the previously mentioned models (Table 6).

**Table 6:** Shows the different kinetic models for different NPs

Sample	Acidic buffer	Model name	Basic buffer	Model name	Reference
Chitosan/polyvinylpyr-rolidone	0.9604	Higuchi	0.9753	Korsmeyer–Peppas	61
Chitosan/graphene oxide	0.9127	Korsmeyer–Peppas	0.9503	Korsmeyer–Peppas	62
CeO_2_	0.9944	Higuchi	0.9834	Korsmeyer–Peppas	This work

### 3.5 Photocatalytic activity green synthesized CeO_2_-NPs

**Fig. 16:**
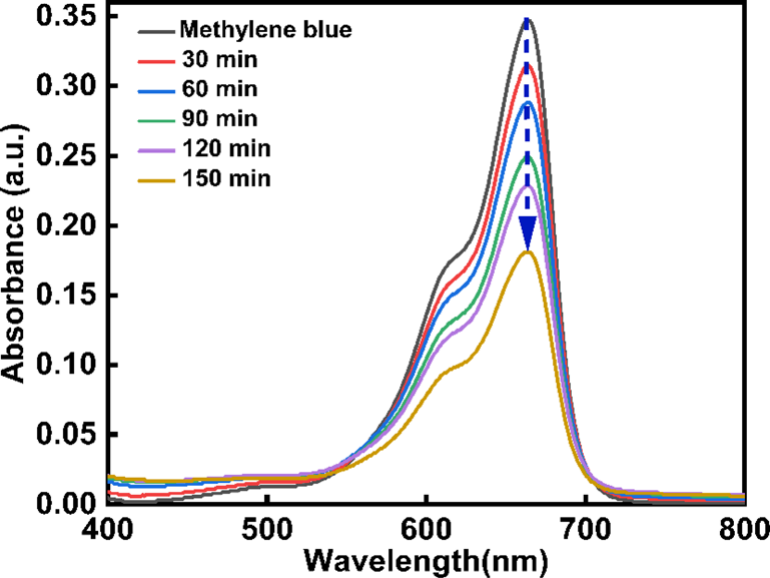
Degradation of methylene blue dye by CeO_2_-NPs under UV irradiation.

One of the most popular industrial dyes is methylene blue (MB). Furthermore, MB is frequently employed in analytical chemistry as a redox indicator. This substance's solutions are blue in an oxidizing environment and turn colorless when they come into contact with a reducing agent.^63^ MB poses a serious risk to both human health and the environment because it is poisonous, carcinogenic, and non-biodegradable. It becomes a health hazard to humans and other living things when it is released into natural water sources. For this reason, an effective and ecologically friendly method of extracting MB from wastewater^64,65^ must be developed. In this work, the degradation of MB dye under UV light was used to explain the photocatalytic activity of CeO_2_-NPs. Fig. 16 displays the CeO_2_-NPs degradation activity against MB. At regular intervals of 30, 60, 90, 120, and 150 minutes, the MB solution's absorption spectra were recorded. Also, the rate of discoloration resulting from variations in the intensity of the absorption peak at 664 nm was examined. The CeO_2_-NPs exhibit a significant catalytic efficiency, resulting in a 56.77% degradation of the dye after 150 minutes, as evidenced by the presence of a band at 664 nm.

### 3.6 Mechanism of photocatalytic decay

**Fig. 17:**
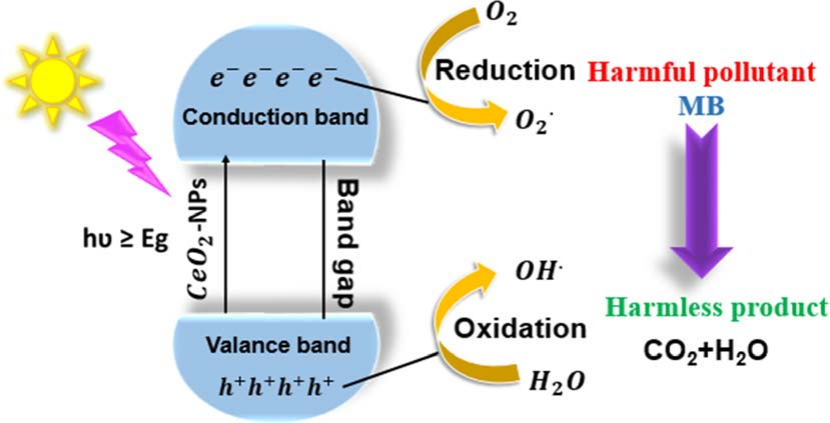
Mechanism of photodegradation of MB dyes using CeO_2_-NPs.

The photocatalytic activity of green synthesized CeO_2_-NPs has been depicted in Fig. 17. Upon exposure to UV light, CeO_2_-NPs, having either greater than or equal to the semiconductor's band gap energy (*E*_g_), absorb energy (*hν*), and the electrons (e^−^) from the valence band absorb energy and move to the conduction band, leaving a hole (h^+^) in the valence band. Consequently, e^−^/h^+^ pairs are originated and the carrier is moved outside.

Superoxide radicals (O_2_^−^) are created when electrons oxidize attractive oxygen, forming hydroxyl radicals (˙OH) that form the pore water molecules ions. Significantly affecting the degradation of the dye was the formation of apertures and electrons in the valence and conduction bands. The following is the photocatalysis reaction mechanism:^42,66^

When photons were absorbed by CeO_2_-NPs, electrons were excited. The generation of hole-pairs can occur within CeO_2_-NPs, as described in eqn (19).19
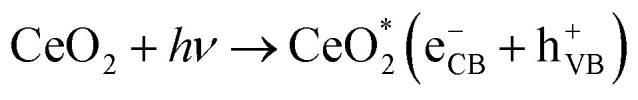
where 
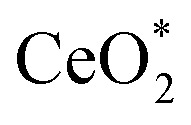
 was the excited state of CeO_2_, e_CB_^−^ was a photoexcited electron in the conduction band, and h_VB_^+^ was a photogenerated hole in the valence band.^63^

20 CeO_2_(h_CB_^+^) + H_2_O → OH˙ + H^+^

21 CeO_2_(e_CB_^−^) + O_2_ → O_2_˙^−^

22 O_2_˙^−^ + MBdye → CO_2_ + H_2_O

23 OH˙ + MBdye → CO_2_ + H_2_O

The oxygen and water molecules are adsorbed on the photocatalyst's surface. These molecules react with the electron–hole pairs (eqn (20) and (21)) to produce the unstable hydroxyl radicals (OH˙) and superoxide ions (O_2_˙^−^), which oxidize the organic pollutants into inorganic compounds (eqn (22) and (23)).

### 3.7 Antifungal effect of CeO_2_-NPs

**Fig. 18:**
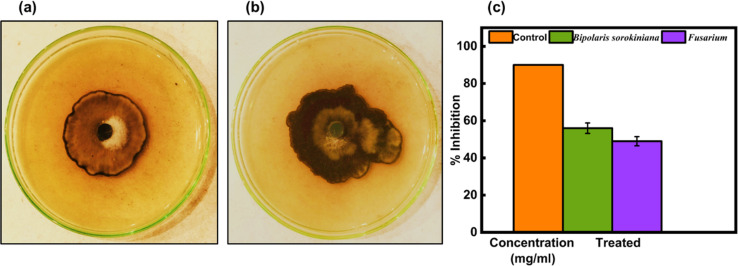
*In vitro* control of (a) *B. sorokiniana* and (b) *Fusarium* using the green synthesized CeO_2_-NPs. (c) Mycelial growth inhibition against fungi.

Green-synthesized CeO_2_-NPs were screened against fungus strains such as *B. sorokiniana* and *Fusarium* using the well diffusion method. The outcomes have been shown in Fig. 18(a and b), and the PDA agar plates were treated with 1.35 mg mL^−1^ of CeO_2_-NPs. The zone of inhibition results in Fig. 18(c) revealed that CeO_2_-NPs were effective against *B. sorokiniana* (56%) and *Fusarium* (49%).

## 4. Conclusions

This study was actually focused on the green synthesis of CeO_2_-NPs using *Oroxylum indicum* fruit extract, and several analytical techniques, including XRD, SEM, EDX, UV-vis, FTIR, and VSM, certainly confirmed the formation of NPs with a particle size of 23.58 nm. The NPs were used as nano carriers for drug delivery systems that showed proficient activity along with synergistic effects in antioxidant and antifungal activity. Moreover, the synthesized NPs were demonstrated to be photo-catalysts for the degradation of MB in the presence of UV light, suggesting the further use of this catalyst to eliminate toxic dye from aqueous medium.

## Data availability

The data that support the findings of this study are available on request from the corresponding author. The data are not publicly available due to restrictions *e.g.* their containing information that could compromise the privacy of research participants.

## Author contributions

Jannatul Mim: validation, investigation, formal analysis, writing – original draft. Mst. Sabiha Sultana: investigation, validation, writing – review & editing. Palash Kumar Dhar: visualization, writing – review & editing. Sagar Kumar Dutta: conceptualization, methodology, writing – review & editing, visualization, software, supervision.

## Conflicts of interest

There are no conflicts to declare.
